# The Effect of Limited Ankle Dorsiflexion During Emergency Stop—Jump Movements on Lower Limb Biomechanics

**DOI:** 10.1002/jfa2.70165

**Published:** 2026-06-13

**Authors:** Yijing Zhou, Nan Yang, Qiaoqiao Wang, Yuan Wu, Haitao Shi, Si Zhang, Shuang Ren, Hongshi Huang

**Affiliations:** ^1^ Department of Sports Medicine Beijing Key Laboratory of Research and Translation for Drugs and Medical Devices in Precision Diagnosis and Treatment of Sports Injuries Engineering Research Center of Sports Trauma Treatment Technology and Devices, Ministry of Education Peking University Third Hospital Institute of Sports Medicine of Peking University Beijing China; ^2^ Tianjin Key Laboratory of Exercise Physiology and Sports Medicine Institute of Sport Exercise & Health Tianjin University of Sport Tianjin China

**Keywords:** biomechanics, landing, limited ankle dorsiflexion, stiffness, stop‐jump

## Abstract

**Objective:**

The stop‐jump task is a key movement in sports such as basketball and volleyball, with landing biomechanics closely linked to injury risk. Restricted ankle dorsiflexion (DF) alters lower‐extremity mechanics and increases the risk of lower‐limb joint injuries; however, its effects on the full stop‐jump cycle remain unclear. This study examined how restricted DF influences lower‐extremity biomechanics during the stop‐jump task and explored the mechanisms underlying a stiff landing strategy and its potential injury risks.

**Methods:**

Participants underwent static ankle dorsiflexion range of motion (DF ROM) testing and functional squat assessment. Eligible participants completed walking and stop‐jump tasks, with biomechanical data collected synchronously using motion capture and force plates. Based on peak ankle dorsiflexion angle during walking, 44 participants were categorized into restricted (RADF, *n* = 22) and unrestricted (unRADF, *n* = 22) groups. Group differences in lower‐extremity biomechanics across stop‐jump phases were analyzed using ANCOVA with sex as a covariate, with post hoc power reported.

**Results:**

The RADF group demonstrated significantly greater peak vertical ground reaction force (GRF) and posterior loading rates (LRs) compared with the unRADF group (*p* < 0.05). During the horizontal landing phase, ankle plantarflexion and internal rotation angles were significantly increased in the RADF group (*p* < 0.05). At the moment of peak GRF during the vertical landing phase, the RADF group exhibited significantly reduced ankle dorsiflexion angle (*p* < 0.001) and significantly greater knee abduction angle (*p* < 0.05). Additionally, leg stiffness was significantly higher in the RADF group during this phase (*p* < 0.05).

**Conclusion:**

Individuals with restricted DF adopt a stiff landing strategy during stop‐jump, with increased impact loads and abnormal joint kinetics that may impair shock absorption and elevate lower‐extremity injury risk.

## 1 Introduction

The stop‐jump task is a common movement frequently performed in sports such as basketball and volleyball. It reflects an athlete's integrated ability to rapidly decelerate, change direction, and explosively initiate a jump under high‐speed conditions. This complex movement consists of an approach run, a sudden stop (horizontal landing phase), and subsequent take‐off and vertical landing phases [1, 2]. Different landing strategies during the landing phase are closely associated with impact forces and knee joint loading [1]. A stiff landing strategy is characterized by reduced flexion of the lower‐extremity joints, which results in greater ground reaction force (GRF) and increased anterior shear forces at the knee. Consequently, this leads to substantially elevated loading on the quadriceps, patellar tendon, and ACL [3]. Therefore, optimal landing technique not only directly influences the quality of subsequent movement execution but is also closely associated with the risk of lower‐extremity sports injuries [4].

Ankle dorsiflexion range of motion (DF ROM) is essential for the performance of functional activities such as walking, jogging, landing, and stair negotiation. Restricted functional ankle dorsiflexion (DF) has been associated with altered movement patterns. DF ROM assessed during functional tasks provides a more comprehensive reflection of ankle joint function under loading conditions, neuromuscular control, and its relationship with lower‐extremity kinetics. Therefore, compared with passive ROM assessments, functional DF ROM is of greater clinical relevance for evaluating movement performance and injury risk [5, 6, 7, 8]. Previous studies have demonstrated that restricted DF alters lower‐extremity biomechanical patterns during jump‐landing by inducing proximal compensatory mechanisms, thereby increasing the risk of sports‐related injuries [1, 9, 10, 11]. Restricted DF alters the way kinetic energy is absorbed during landing, resulting in proximal energy transfer through biarticular muscles and subsequently inducing compensatory strategies at the hip and knee joints. These compensations are characterized by reduced joint range of motion during landing, increased GRF and loading rates (LRs), as well as a delayed time to peak GRF [8, 11, 12, 13, 14, 15, 16, 17]. Collectively, these biomechanical alterations suggest that individuals with restricted DF tend to adopt a “stiff” landing strategy, which is strongly associated with an increased risk of patellofemoral pain syndrome (PFPS) and anterior cruciate ligament (ACL) injuries [18, 19]. However, existing studies have largely focused on descriptive kinematic and kinetic outcomes, with limited investigation into the underlying mechanical mechanisms of the stiff landing strategy. Hatefi et al. [20] reported a moderate positive correlation between ankle DF ROM and ankle dorsiflexion stiffness. In addition, previous research has demonstrated that reduced frontal‐plane hip stiffness is significantly associated with kinematic abnormalities related to dynamic knee valgus, a potential biomechanical marker for landing‐related injuries such as ACL injury [21]. Stiffness reflects the capacity to resist GRF and includes vertical stiffness, leg stiffness, and joint stiffness [22], all of which play a critical role in movement stability. Excessive stiffness has been associated with bony injuries, whereas insufficient stiffness is linked to an increased risk of soft tissue injuries [23]. Previous studies have adopted varying definitions of ankle joint stiffness, including static stiffness measures [22]. In contrast, dynamic joint stiffness (DJS) is defined as the resistance generated by muscles spanning the joint, together with passive constraints (e.g., ligaments), during segmental displacement under external moments [24]. As such, DJS provides a more comprehensive representation of overall joint functional status during dynamic tasks such as the stop‐jump maneuver. Furthermore, variations in leg and joint stiffness may provide valuable insight into motor control responses to neuromuscular factors, including fatigue‐induced alterations in neural and muscular function [25]. Existing studies have preliminarily suggested that restricted DF during landing leads to compensatory increases in knee and hip extension to attenuate impact, manifested as increased landing stiffness and elevated GRF and LRs. However, such studies have predominantly focused on isolated vertical landing tasks, emphasizing the kinematic and kinetic characteristics of landing itself. In contrast, ACL injuries often occur during more complex, multi‐phase movements. The stop‐jump task, which incorporates both horizontal and vertical landing phases, exhibits distinct mechanical characteristics across its different phases. How DF restriction influences joint stiffness and phase‐specific mechanical control strategies in such a complex task remains unclear. Particularly at the instant of peak GRF, when the interaction force between the body and the ground reaches its maximum, mechanical loads on joints and soft tissues are greatest, making this moment critical for injury occurrence [26, 27, 28, 29, 30]. To date, limited research has investigated how restricted DF affects multi‐joint coordination and load response characteristics of the lower extremity at this critical instant. Accordingly, the present study proposes the following hypothesis:

1. During the vertical landing phase, restricted DF results in a higher peak vertical GRF, peak posterior GRF, and increased LRs.
2. During the stop (horizontal landing phase), restricted DF will lead to reduced ankle joint angles, increased knee valgus range of motion, and greater knee extension moment.
3. At the instants of peak vertical and posterior GRF, restricted DF will cause significantly reduced ankle joint ROM, decreased sagittal plane ROM at the knee and hip, increased knee valgus angle, and significantly greater knee extension and hip extension moments.
4. Restricted DF leads to increased leg stiffness during the vertical landing phase, as well as greater frontal‐plane stiffness at the ankle and hip joints, and increased transverse‐and frontal‐plane stiffness at the knee joint.

## 2 Materials and Method

### 2.1 Participants

This cross‐sectional study recruited 44 participants (37 males and 7 females). The study protocol was approved by the Ethics Committee of Peking University Third Hospital (Approval No. M2023360). All participants provided written informed consent after receiving a detailed explanation of the experimental procedures.The inclusion criteria were as follows: (1) a normal body mass index (18.5–24.9 kg/m^2^); (2) no history of neurological disorders; (3) absence of musculoskeletal conditions that restricted physical activity within the previous 6 months; and (4) no prior lower‐limb or pelvic surgery or injury. All participants exhibited sufficient physical ability to complete at least three walking and stop‐jump trials and reported no pain or discomfort during testing. Participants were publicly recruited from the Sports Medicine Outpatient Clinic of Peking University Third Hospital. Prior to inclusion, all participants underwent passive ankle dorsiflexion measurements in both the supine knee‐extended position (< 10°) [31, 32, 33]. In addition, a squat test was performed, with restriction defined as the inability to fully flex the knee or forced heel lift when performing a deep squat with feet positioned shoulder‐width apart. Participants exhibiting such restrictions were included in the subsequent experimental procedures of this study.

Previous studies have evaluated DF ROM using both static measurements (e.g., with goniometers or inclinometers) under weight‐bearing and non‐weight‐bearing conditions [8, 14, 34, 35, 36, 37]as well as dynamic measurements during functional tasks using motion capture systems [38]. These approaches consistently use the peak DF angle as the primary metric. Notably, the peak DF ROM measured during functional tasks offers a more comprehensive assessment of athletic performance and injury risk. Gao et al. [7] proposed 9.03° during the stance phase of walking as a diagnostic cut‐off value for functional restricted DF. Consequently, the present study adopted this criterion to identify restricted ankle mobility, enabling a more accurate evaluation of DF ROM insufficiency during functional movement. The sample size was calculated in advance using G*Power software, with an alpha level of 0.05, a statistical power of 80%, and an expected effect size of 1.0. Based on the peak DF ROM measured during walking in one randomly selected limb, participants were assigned into two groups: those with a peak DF ROM < 9.03° were assigned to the restricted ankle dorsiflexion (RADF) group (*n* = 22), whereas the remaining participants comprised the unrestricted (unRADF) group (*n* = 22).

### 2.2 Data Collection

**FIGURE 1:**
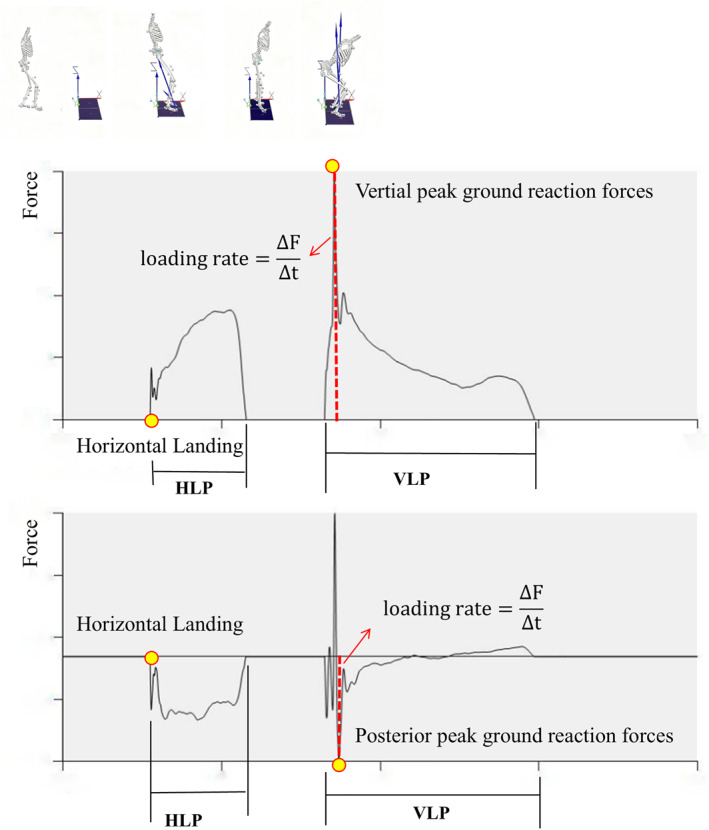
Stop‐jump task sequence: run‐up, two‐foot simultaneous landing, and subsequent vertical jump and landing. HLP, horizontal landing phase; VLP, vertical landing phase.

This study utilized an eight‐camera infrared high‐speed motion capture system (Vicon, T40) to collect static and three‐dimensional dynamic kinematic data at a sampling frequency of 100 Hz. Simultaneously, kinetic data were recorded at 1000 Hz using two force plates (AMTI, BP400600), synchronized via a synchronization device (AMTI, GEN5). Participants wore athletic shorts to reveal their lower extremities and the area beneath the waist. Reflective markers were attached to anatomical landmarks, and data were processed using the Plug‐in Gait model, following international standards. Prior to formal testing, participants familiarized themselves with the experimental procedures and movement requirements. During the static calibration phase, participants positioned themselves in the center of the force platforms with their feet shoulder‐width apart, arms relaxed at their sides, and ankles in a neutral alignment. Three static data collections were performed to establish individual skeletal coordinate systems.Subsequently dynamic testing included two tasks: (1) Walking: Participants walked at a self‐selected comfortable speed. Five valid trials were collected per participant, and the average values were used for subsequent analysis. (2) Stop‐Jump Task (Figure 1): Participants performed a maximal run‐up, achieved peak velocity, and landed simultaneously with both feet on separate force platforms. After a brief stabilization phase, they performed a maximal vertical jump followed by landing. Each participant successfully completed three valid trials that met the following criteria: ①Both feet landed simultaneously on the platforms to ensure symmetrical loading; ②Both the initial stop and subsequent landing after the jump occurred entirely within the force platform boundaries. Trials that failed to meet these criteria were discarded and repeated. All movements were performed barefoot, and the interval between two consecutive trials was determined based on the participant's perception of fatigue, ensuring that they did not experience undue exertion.

### 2.3 Data Processing

Lower‐limb biomechanical analyses were performed in Visual 3D (C‐Motion, Germantown, MD; v6.00.18) based on inverse dynamics principles. Subject‐specific skeletal models were established, and kinematic data were filtered using a 12 Hz low‐pass Butterworth filter. Following standard procedures [39, 40, 41], GRF signals were filtered at 50 Hz. The calculated knee joint moments were internal (endogenous) extension moments. Joint moments were normalized to body height and mass, whereas GRF values were expressed relative to body weight and gravitational acceleration to control for anthropometric variability. The filtered datasets were imported into MATLAB (MathWorks Inc.) for further reduction and analysis with custom‐written routines. The variables analyzed included peak three‐dimensional GRF and LRs, with the instant of the peak GRF identified as the critical moment of injury occurrence. Analyzing the joint angles and moments of the lower extremities at the moment of the peak GRF helps to clarify whether the two groups of subjects adopted different movement strategies during the stop‐jump maneuver, thereby further identifying potential injury risks.

#### 2.3.1 Calculation of Leg and Joint Stiffness

Stiffness is defined as the ratio of the change in force to the change in length. In the human body, stiffness reflects the ability to resist deformation induced by GRF [23, 42]. In this study, a torsional‐spring model was used to calculate dynamic joint stiffness during the eccentric phase of the stop‐jump maneuver in both horizontal and vertical landing conditions [42, 43]. The eccentric phase was defined as the period from initial contact with the force plate (vertical GRF > 10 N) to the time of maximum knee flexion. Joint stiffness (Nm/deg/kg) was calculated for each joint using the following formula:

Kjoint(Nm/deg/kg)=ΔMjoint/Δθjoint.

In the formula, ΔMjoint represents the change in joint torque from initial foot contact with the force plate to the point of maximum knee flexion, and Δθjoint represents the change in joint angular displacement over the same period. All joint stiffness variables were normalized by subject body weight [44].

Additionally, leg stiffness (BW/m) was defined as the ratio of the peak GRF to the peak vertical displacement of the center of mass during the stop‐jump task, and was calculated using the following formula [45, 46]:

$$\text{Kleg}\;\left(\frac{N}{m}\right)=\frac{\text{peak}\;\text{vGRF}}{\Delta \text{LCOM}}.$$

In the formula, peak vGRF represents the peak vertical ground reaction force, and ΔLCOM represents the vertical displacement of the center of mass from initial foot contact with the force plate to the moment of maximum knee flexion. All lower extremity stiffness variables were normalized by subject body weight.

### 2.4 Statistical Analysis

Before the primary analysis, independent‐sample t‐tests were used to compare demographic variables among the two groups for statistically significant differences. The Shapiro‐Wilk test was first employed to test the normality of the quantitative data. For data following a normal distribution, results were presented as mean ± standard deviation (Mean ± SD); for data not following a normal distribution, nonparametric tests were used. Given that sex may influence both the kinetic and kinematic outcome measures of the stop‐jump task, it was included as a covariate in the analysis. Between‐group comparisons were performed using analysis of covariance (ANCOVA), with group (RADF, unRADF group) as the fixed variable and sex as the covariate. All statistical analyses were conducted using SPSS software (version 27.0), and a *p*‐value < 0.05 was considered statistically significant.

## 3 Results

### 3.1 Subject Information

Descriptive results regarding demographic data and biomechanical data at peak DF ROM are presented in Table 1. The study enrolled 44 participants (37 males and 7 females). Based on the peak DF ROM measured during the stance phase of gait, participants were classified into RADF group (< 9.03°, *n* = 22; 20 males) and unRADF group (≥ 9.03°, *n* = 22; 17 males). Age, height, and body weight did not differ significantly between groups (*p* > 0.05). However, the peak DF ROM was significantly lower in the RADF group compared with the unRADF group (*p* < 0.01).

**TABLE 1:** Participant characteristics.

Variables	unRADF group (SD)	RADF group (SD)	*t*‐value	*p*‐value
Height (cm)	172.34 (8.75)	175.43 (7.76)	1.25	0.22
Body mass (kg)	74.32 (13.25)	73.75 (13.85)	−0.14	0.89
BMI (kg/m^2^)	24.85 (2.93)	23.79 (3.01)	−1.18	0.25
Age (years)	30.77 (12.68)	29.45 (11.88)	−0.36	0.72
Peak DF ROM (°)	12.17 (2.01)	6.03 (2.03)	−10.06	< 0.01*

Abbreviations: CI, Confidence Interval; SD, standard deviation.

* *p* < 0.05.

### 3.2 Peak Ground Reaction Forces and Loading Rates

Using sex as a covariate, the ANCOVA results for peak vertical GRF, peak posterior GRF, LRs, and time to LRs during the stop‐jump maneuver are presented in Table 2. The parameters that were significantly greater in the RADF group than in the unRADF group were as follows: peak vertical GRF (RADF group: 2.35 ± 0.10 BW, unRADF group: 1.83 ± 0.10 BW, *p* < 0.05); vertical LRs (RADF group: 205.91 ± 1.70 BW/s, unRADF group: 150.41 ± 1.70 BW/s, *p* < 0.05); and posterior LRs (RADF group: 46.40 ± 3.80 BW/s, unRADF group: 30.71 ± 3.74 BW/s, *p* < 0.05). No significant differences were observed for the other parameters.

**TABLE 2:** Peak vertical ground reaction force, peak loading rate, and time to peak loading rate.

Variable	RADF	unRADF	RADF	Un‐RADF	*p*‐value	Partial *η* ^2^	Post‐hoc power
	Mean (SD)		Adjust mean (SE)				
Peak vertical GRF (BW)	2.33 (0.48)	1.85 (0.46)	2.29 (0.09)	1.89 (0.09)	0.004*	0.184	0.843
Peak posterior GRF (BW)	0.24 (0.88)	0.28 (0.09)	0.23 (0.19)	0.28 (0.9)	0.102	0.064	0.372
Vertical LR (BW/s)	205.91 (81.25)	150.42 (60.05)	202.11 (14.90)	154.21 (14.90)	0.030*	0.110	0.595
Posterior LR (BW/s)	45.73 (18.67)	31.64 (16.29)	45.00 (3.72)	32.37 (3.72)	0.022*	0.122	0.643
Timing of vertical LR (s)	1.56 (0.30)	1.68 (0.26)	1.54 (0.06)	1.69 (0.06)	0.072	0.077	0.438
Timing of posterior LR (s)	1.58 (0.28)	1.71 (0.25)	1.57 (0.06)	1.72 (0.06)	0.065	0.081	0.456

Abbreviations: SD, standard deviation; SE, Standard Error.

* *p* < 0.05.

### 3.3 Horizontal Landing Phase (Stop Phase)

**FIGURE 2:**
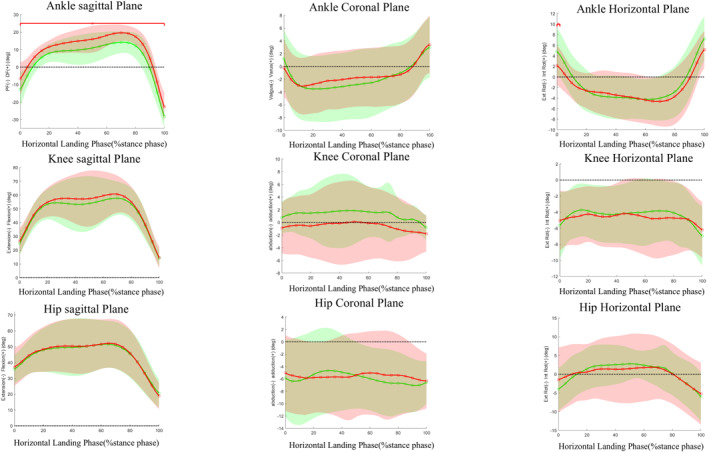
Variations in joint motion angles during horizontal landing phase in the two groups of subjects. *x*‐axis: Normalized stance phase (%); *y*‐axis: joint angles (°). The RADF group is shown in green, and the unRADF group in red. Significant differences are highlighted by red horizontal lines. DF, dorsiflexion; Ext Rot, external rotation; Int Rot, internal rotation; PF, plantarflexion.

**FIGURE 3:**
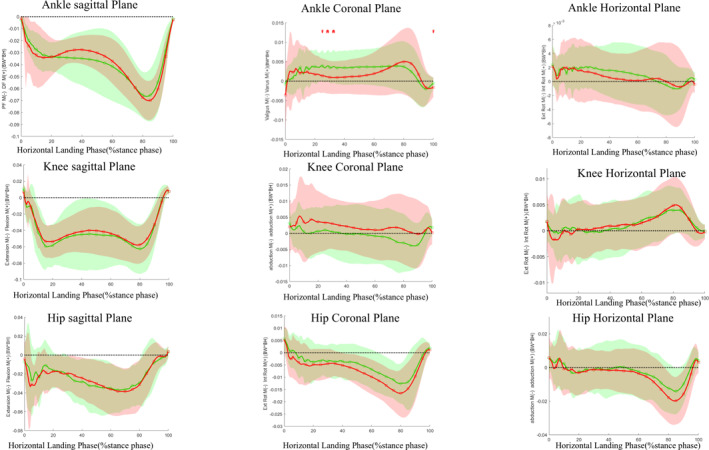
Variations in moments during horizontal landing phase in the two groups of subjects. *x*‐axis: Normalized stance phase (%); *y*‐axis: joint moments. The RADF group is shown in green, and the unRADF group in red. Significant effects are indicated by red horizontal lines. BW, body weight; BW*BH, body weight*body height; Ext M, extension moment; ExtR M, external rotation moment; Fle M, flexion moment; IntR M, internal rotation moment.

Using sex as a covariate, the ANCOVA results showed that during the horizontal landing phase of the stop‐jump maneuver, the RADF group exhibited a significant increase in ankle internal rotation angle compared with the unRADF group (4.85 ± 0.85° vs. 2.36 ± 0.85°, *p* < 0.05). No significant differences were observed for the other parameters (Table 3, Figures 2 and 3).

**TABLE 3:** Biomechanical parameters at stop phase.

Variable	RADF	unRADF	RADF	Un‐RADF	*p*‐value	Partial *η* ^2^	Post‐hoc power
	Mean (SD)		Adjust mean (SE)				
Ankle dorsiflexion (°)	−12.73 (10.00)	−6.68 (9.65)	−12.91 (2.13)	−6.51 (2.13)	0.041*	0.098	0.539
Ankle Eversion (°)	1.76 (4.40)	−0.15 (4.60)	1.91 (0.97)	−0.30 (0.97)	0.116	0.059	0.347
Ankle internal rotation (°)	4.97 (4.18)	2.24 (3.89)	5.15 (0.85)	2.06 (0.85)	0.015*	0.36	0.699
Knee flexion (°)	25.14 (8.74)	26.70 (9.67)	25.07 (2.01)	26.78 (2.01)	0.552	0.009	0.090
Knee abduction (°)	0.77 (2.63)	−0.87 (4.11)	0.54 (0.70)	−0.64 (0.70)	0.245	0.033	0.210
Hip flexion (°)	35.83 (9.12)	37.25 (12.13)	35.96 (2.33)	37.12 (2.33)	0.729	0.003	0.063
Knee flexion moment (BW*BH)	0.011 (0.006)	0.007 (0.011)	0.011 (0.002)	0.007 (0.002)	0.144	0.051	0.308
Hip extension moment (BW*BH)	−0.006 (0.013)	−0.006 (0.015)	−0.006 (0.003)	−0.006 (0.003)	0.897	< 0.001	0.052

Abbreviations: SD, standard deviation; SE, Standard Error.

* *p* < 0.05.

### 3.4 Lower Extremity Biomechanics at Peak Vertical GRF

**FIGURE 4:**
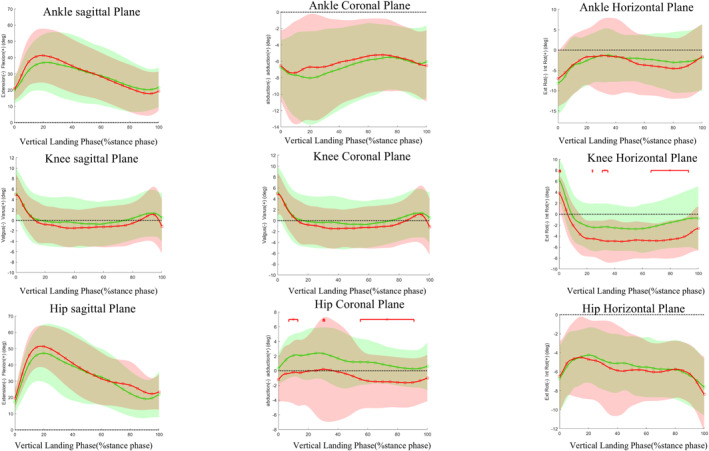
Variations in joint motion angles during vertical landing phase in the two groups of subjects. *x*‐axis: Normalized stance phase (%); *y*‐axis: joint angles (°). The RADF group is shown in green, and the unRADF group in red. Significant differences are highlighted by red horizontal lines. DF, dorsiflexion; Ext Rot, external rotation; Int Rot, internal rotation; PF, plantarflexion.

**FIGURE 5:**
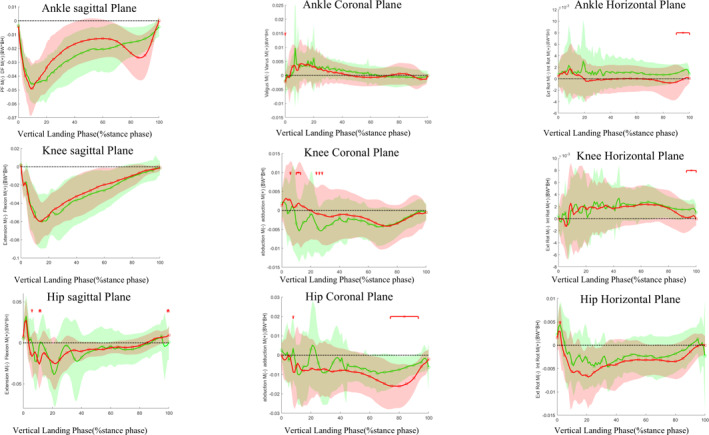
The variations of moments during vertical landing phase in the two groups of subjects. *x*‐axis: Normalized stance phase (%); *y*‐axis: joint moments. The RADF group is shown in green, and the unRADF group in red. Significant effects are indicated by red horizontal lines. BW, body weight; BW*BH, body weight*body height; Ext M, extension moment; ExtR M, external rotation moment; Fle M, flexion moment; IntR M, internal rotation moment.

Using sex as a covariate, the ANCOVA results showed that at the instant of the peak vertical ground reaction force during the vertical landing phase of the stop‐jump maneuver, the parameters that were significantly smaller in the RADF group than in the unRADF group were: ankle dorsiflexion angle (RADF group: 11.56 ± 0.77°, unRADF group: 15.96 ± 0.77°, *p* < 0.05); and the parameters that were significantly greater in the RADF group than in the unRADF group were: knee abduction angle (RADF group: 2.26 ± 0.84°, unRADF group: −0.34 ± 0.84°, *p* < 0.05). No significant differences were observed for the remaining parameters (Table 4, Figures 4 and 5).

**TABLE 4:** Biomechanical parameters at peak vertical GRF.

Variable	RADF	unRADF	RADF	unRADF	*p*‐value	Partial *η* ^2^	Post‐hoc power
	Mean (SD)		Adjust mean (SE)				
Ankle dorsiflexion (°)	11.56 (3.08)	15.95 (3.99)	11.55 (0.78)	15.96 (0.78)	< 0.001*	0.280	0.973
Knee flexion (°)	42.75 (11.76)	41.70 (9.56)	42.34 (2.29)	42.10 (2.29)	0.941	< 0.001	0.051
Knee abduction (°)	2.21 (2.91)	−0.29 (4.71)	2.29 (0.85)	−0.37 (0.85)	0.034*	0.105	0.573
Hip flexion (°)	34.25 (12.49)	33.98 (11.69)	33.78 (2.58)	34.45 (2.58)	0.856	0.001	0.054
Knee flexion moment (BW*BH)	−0.075 (0.027)	−0.073 (0.033)	−0.074 (0.006)	−0.075 (0.006)	0.948	< 0.001	0.050
Hip extension moment (BW*BH)	−0.006 (0.120)	0.018 (0.047)	−0.004 (0.020)	0.016 (0.020)	0.485	0.012	0.106

Abbreviations: SD, standard deviation; SE, Standard Error.

* *p* < 0.05.

### 3.5 Lower Extremity Biomechanics at Peak Posterior GRF

Using sex as a covariate, the ANCOVA results showed that at the instant of the peak vertical ground reaction force during the vertical landing phase of the stop‐jump maneuver, the parameter that was significantly greater in the RADF group than in the non‐RADF group was the knee adduction angle (RADF group: 1.60 ± 0.58°, unRADF group: −1.05 ± 0.58°, *p* < 0.05). No significant differences were observed for the remaining parameters (Table 5, Figures 4 and 5).

**TABLE 5:** Biomechanical parameters at peak posterior GRF.

Variable	RADF	unRADF	RADF	unRADF	*p*‐value	Partial *η* ^2^	Post‐hoc power
	Mean (SD)		Adjust mean (SE)				
Ankle dorsiflexion (°)	5.44 (1.00)	10.36 (7.90)	5.32 (1.96)	10.48 (1.96)	0.072	0.077	0.437
Knee flexion (°)	41.17 (13.52)	45.05 (10.60)	40.81 (2.61)	45.41 (2.61)	0.225	0.036	0.226
Knee abduction (°)	1.52 (3.30)	−0.97 (4.59)	1.20 (0.79)	−0.65 (0.79)	0.107	0.62	0.363
Hip flexion (°)	34.37 (12.62)	37.26 (11.61)	34.02 (2.61)	37.61 (2.61)	0.340	0.022	0.156
Knee flexion moment (BW*BH)	−0.724 (0.038)	−0.071 (0.018)	−0.072 (0.006)	−0.071 (0.006)	0.927	< 0.001	0.051
Hip extension moment (BW*BH)	−0.006 (0.120)	0.018 (0.047)	−0.004 (0.020)	0.016 (0.020)	0.485	0.012	0.106

Abbreviations: SD, standard deviation; SE, Standard Error.

### 3.6 Leg Stiffness and Joint Stiffness

Using sex as a covariate, the ANCOVA results showed that during the vertical landing phase of the stop‐jump maneuver, lower extremity joint stiffness was significantly higher in the RADF group than in the unRADF group (RADF group: 23.50 ± 1.75 BW/m, unRADF group: 18.10 ± 1.75 BW/m, *p* < 0.05). No significant difference was observed during the horizontal landing phase (Table 6).

**TABLE 6:** Leg stiffness (BW/m).

Variable	RADF	unRADF	RADF	unRADF	*p*‐value	Partial *η* ^2^	Post‐hoc power
	Mean (SD)		Adjust mean (SE)				
Leg stiffness—Horizontal landing phase	41.65 (18.51)	41.95 (23.02)	41.44 (5.28)	42.16 (5.28)	0.923	< 0.001	0.051
Leg stiffness—Vertical landing phase	22.90 (8.48)	18.70 (8.43)	23.50 (1.75)	18.10 (1.75)	0.037*	0.107	0.558

Abbreviations: SD, standard deviation; SE, Standard Error.

* *p* < 0.05.

### 3.7 Joint Stiffness of Horizontal and Vertical Landing Phase

Using sex as a covariate, the ANCOVA results showed no significant differences in any of the joint stiffness parameters during the horizontal and vertical landing phase of the stop‐jump maneuver (*p* > 0.05, Table 7).

**TABLE 7:** Jiont stiffness of horizontal and vertical landing phase (Nm/deg/kg).

Variable	RADF	unRADF	RADF	unRADF	*p*‐value	Partial *η* ^2^	Post‐hoc Power
	Mean (SD)		Adjust mean (SE)				
Horizontal landing phase							
Ankle stiffness—Frontal plane	0.15 (0.73)	0.15 (0.92)	0.15 (0.18)	0.15 (0.18)	0.815	0.001	0.056
Ankle stiffness—Transverse plane	0.20 (0.36)	0.10 (0.59)	0.22 (0.10)	0.08 (0.10)	0.358	0.021	0.149
Ankle stiffness—Rotation plane	0.01 (0.08)	0.03 (0.19)	0.003 (0.31)	0.04 (0.31)	0.422	0.016	0.142
Knee stiffness—Frontal plane	0.17 (0.08)	0.14 (0.05)	0.17 (0.14)	0.14 (0.14)	0.087	0.070	0.403
Knee stiffness—Transverse plane	0.16 (0.15)	0.12 (0.31)	0.17 (0.07)	0.11 (0.07)	0.559	0.015	0.088
Hip stiffness—Frontal plane	0.15 (0.64)	0.13 (0.16)	0.14 (0.23)	0.14 (0.23)	0.992	< 0.001	0.050
Vertical landing phase							
Ankle stiffness—Frontal plane	0.08 (0.39)	0.07 (0.32)	0.08 (0.01)	0.07 (0.01)	0.285	0.028	0.185
Knee stiffness—Frontal plane	0.16 (0.69)	0.12 (0.44)	0.16 (0.01)	0.13 (0.01)	0.109	0.061	0.359
Knee stiffness—Transverse plane	0.16 (0.09)	0.01 (0.40)	0.16 (0.08)	0.01 (0.08)	0.150	0.075	0.298
Hip stiffness—Frontal plane	0.04 (0.07)	0.07 (0.06)	0.04 (0.02)	0.07 (0.02)	0.150	0.050	0.300

Abbreviations: SD, standard deviation; SE, Standard Error.

## 4 Discussion

The present study aimed to investigate lower extremity biomechanical characteristics and potential mechanisms in participants with RADF compared with those without RADF during different phases of the stop‐jump maneuver, thereby further improving the understanding of how restricted DF affects lower extremity biomechanics and joint stiffness throughout the stop‐jump cycle. Our results were generally consistent with our hypotheses. During the stop‐jump maneuver, the RADF group exhibited significantly higher peak vertical GRF, vertical and horizontal LRs compared to the unRADF group. Additionally, the RADF group showed a significantly greater ankle internal rotation angle during the horizontal landing phase, whereas during the vertical landing phase, the RADF group showed significantly reduced DF ROM, increased knee adduction angle, and greater leg stiffness.

During the stop‐jump task, the RADF group exhibited significantly higher peak vertical GRF as well as peak vertical and horizontal LRs, with no significant difference in time to peak loading rate. These findings are consistent with previous studies [15, 47], further supporting the critical role of the ankle joint in impact absorption. Restricted DF leads to a stiffer lower extremity movement pattern during landing and reduces impact absorption capacity, thereby increasing the peak vertical ground reaction force and its loading rates. Moreover, previous studies have reported a negative correlation between DF ROM and vertical loading rates, indicating that individuals with restricted DF tend to exhibit higher impact loading rates [15]. During the jump‐landing phase, the ankle plantar flexors contribute 44% of the total muscular work, playing a major role in energy absorption [48]. Restricted DF impairs this cushioning function, resulting in a more rapid rise in impact force over a shorter period, which may lead to localized tissue stress concentration, increases impact loading on the lower extremity joints, and elevates the risk of injury [49]. The present study found no significant difference in time to peak LRs between the two groups. Time to peak loading rate is primarily influenced by initial contact velocity and the inertial properties of the body, which may be less sensitive to restricted DF [50].

The results of this study showed that during the horizontal landing phase, the RADF group exhibited a significantly greater plantarflexion angle. Restricted DF compensates during landing tasks by adjusting the initial contact posture, thereby maintaining sagittal plane joint displacement to absorb the impact of GRF [51, 52]. Furthermore, compared with the unRADF group, the RADF group demonstrated increased ankle internal rotation at initial foot contact. This is associated with reduced extensibility and increased tension of the soleus and gastrocnemius muscles, which may contribute to rearfoot valgus as a compensatory strategy during dynamic tasks, leading to excessive foot internal rotation and abnormal stress concentration in the mid and forefoot regions [53]. Such repetitive mechanical stress may compromise the structural integrity of the medial longitudinal arch by stretching the plantar fascia and surrounding ligamentous structures. Although the RADF group showed a trend toward knee valgus during the stop phase, no significant difference was observed between groups, which contrasts with our initial hypothesis. This result may be attributable to inter‐subject variability in landing strategies: the RADF group exhibited ankle plantarflexion at initial contact (adjusted mean:12.91°) with a standard deviation of ± 10.00°, whereas the unRADF group also showed ankle plantarflexion (adjusted mean:6.51°) with a standard deviation of ± 9.65°. The observed differences in landing patterns, ranging from relatively dorsiflexed initial contact to markedly plantarflexed postures, may substantially influence overall biomechanics and partially obscure differences in knee kinematics between groups. In particular, the initial ankle contact angle may affect the redistribution of lower extremity joint energy dissipation and ground reaction force absorption during landing, thereby potentially increasing the risk of lower extremity injury [54, 55, 56].

The results from both the vertical and horizontal landing phases showed that individuals with restricted DF exhibited greater dynamic ankle dorsiflexion excursion and knee valgus angle at the instant of peak vertical GRF. This finding is consistent with previous evidence [36, 57, 58] showing that greater knee displacement significantly increases ACL loading and injury risk [27, 48, 59, 60, 61]. Therefore, improving DF ROM may help reduce knee injury risk, particularly in sports that frequently involve stop‐jump and landing maneuvers. Although reduced knee flexion during landing is generally associated with increased lower extremity stiffness and injury risk, the present study found no between‐group differences in knee or hip flexion angles or extensor moments at the instants of peak vertical and posterior GRF. This discrepancy from previous studies may be attributable to differences in task demands. Although numerous studies have investigated landing biomechanics [1, 10, 11, 12, 62, 63], the vast majority have focused on vertical drop jumps or used wedged inclines to artificially restrict DF. Different motor tasks impose distinct biomechanical demands, and the inclusion of a vertical jump component in the present experimental protocol may partially explain the differences in findings. Moreover, when individuals exhibit functional restricted DF, they may adopt a variety of compensatory strategies. The present findings suggest that some participants preferentially adjusted their initial landing contact strategy (e.g., adopting a heel‐first contact pattern) rather than relying solely on compensatory movements at the knee and hip. Such variability in compensatory responses may introduce substantial inter‐subject differences, which could partially obscure the direct between‐group effects attributable to restricted DF alone.

Stiffness, including vertical stiffness, leg stiffness, and joint stiffness [22], plays an important role in maintaining joint stability. In biomechanics, stiffness reflects the capacity of the musculoskeletal system to resist deformation while storing and releasing elastic energy during movement. Excessive stiffness may be associated with bony injury, whereas insufficient stiffness may increase the risk of soft tissue injury [23]. In the present study, sex‐adjusted analysis showed that the RADF group exhibited significantly higher lower extremity stiffness during the vertical landing phase of the stop‐jump maneuver compared with the unRADF group, whereas no significant between‐group difference was observed during the horizontal landing phase. The higher leg stiffness observed in the RADF group may reflect an altered cushioning strategy during landing impact. Restricted DF may limit anterior tibial translation and reduce the ankle's capacity for shock absorption during landing, thereby potentially leading to increased lower extremity stiffness to maintain dynamic stability [36, 64]. Thus, higher leg stiffness may indicate that restricted DF is associated with a stiffer landing strategy. However, the difference in leg stiffness was observed only during the vertical landing phase and did not reach statistical significance during the horizontal landing phase. This may be because the vertical landing phase relies primarily on the coordinated action of the hip, knee, and ankle for impact absorption, whereas the horizontal landing phase may rely more on knee joint control [65]. Although a between‐group difference in overall leg stiffness was observed during the vertical landing phase, no significant differences were found in hip, knee, or ankle joint stiffness during landing. This may be explained by the fact that joint stiffness is influenced by multiple factors, including muscle activation and neuromuscular coordination [66, 67]. Despite the absence of significant changes in individual joint stiffness, leg stiffness remained significantly higher, suggesting that landing during the stop‐jump maneuver may depend more on neuromuscular pre‐activation and muscle co‐activation rather than solely on joint torques and joint angles [68, 69, 70, 71, 72]. In contrast, joint stiffness reflects the local mechanical behavior of an individual joint in a specific plane of motion, and its estimation is based on the linear relationship between joint torque and joint angle, which may make it less sensitive to changes in movement strategy.

However, this study has several limitations. The proposed method for identifying functional restricted DF based on peak DF ROM during walking has not been validated against other weight‐bearing or non‐weight‐bearing assessments. Further exploration can be conducted in the future to establish task‐specific criteria for limited ankle dorsiflexion during jump landing. Landing strategies varied among participants, which may substantially affect lower extremity biomechanics. EMG and muscle strength data were not collected, limiting characterization of neuromuscular control and muscle co‐activation. Additionally, the lack of an upper extremity and trunk model prevented analysis of trunk compensation. Future studies should incorporate these factors to better reveal whole‐body biomechanical adaptations in individuals with restricted DF during stop‐jump tasks.

## 5 Conclusions

In conclusion, this study systematically analyzed and compared the lower extremity biomechanical characteristics of individuals with restricted DF by quantifying kinematic, kinetic, and stiffness parameters during both the horizontal and vertical landing phases of a stop‐jump task. Individuals with restricted DF exhibit increased ankle plantar flexion and internal rotation during the horizontal landing phase. During vertical landing, they demonstrate greater lower‐limb stiffness, reduced DF ROM, and increased knee valgus. In addition, peak vertical GRF and LRs are elevated. This movement strategy may reduce shock absorption capacity and increase the risk of lower‐extremity injury. Therefore, training and rehabilitation programs should prioritize restoring DF ROM and improving frontal‐plane stability control.

## Author Contributions

**Yijing Zhou:** conceptualization, data curation, methodology, writing – original draft. **Nan Yang:** investigation, methodology, resources. **Qiaoqiao Wang:** resources. **Yuan Wu:** resources. **Haitao Shi:** resources. **Si Zhang:** resources. **Shuang Ren:** writing – review and editing. **Hongshi Huang:** supervision, writing – review and editing.

## Ethics Statement

Ethics approval was obtained from Peking University Third Hospital Medical Science Research Ethics Committee (M2023360).

## Consent

Informed consent was obtained from all individual participants included in the study.

## Conflicts of Interest

The authors declare no conflicts of interest.

## Data Availability

Research data are not shared.
